# Psychological distress in adults after pediatric kidney replacement therapy

**DOI:** 10.1007/s00467-024-06571-7

**Published:** 2024-11-05

**Authors:** Nora F. Laube, Luzius Mader, Marc-Andrea Heinzelmann, Sandra Hunziker, Gisela Michel, Claudia E. Kuehni, Guido F. Laube

**Affiliations:** 1 Research Group Child & Adolescent Health, Institute of Social and Preventive Medicine, University of Bern, Bern, Switzerland; 2 Childrens University Hospital, Inselspital, University of Bern, Bern, Switzerland; 3 Cancer Registry Bern Solothurn, University of Bern, Bern, Switzerland; 4 Department of Health Sciences and Health Policy, University of Lucerne, Lucerne, Switzerland; 5 Department of Pediatrics, Hospital Baden, Baden, Switzerland; 6 Adolescent Health Research Group, Institute of Social and Preventive Medicine, Swiss Pediatric Renal Registry, University of Bern, Bern, Switzerland

**Keywords:** Psychological distress, Pediatric kidney disease, Kidney replacement therapy, Brief symptom inventory

## Abstract

**Background:**

There is limited information about psychological distress in adults who underwent kidney replacement therapy (KRT) during childhood. This study aimed to describe psychological distress in adults after KRT during childhood in comparison to the Swiss general population and to evaluate associations with sociodemographic and clinical characteristics.

**Methods:**

We sent a questionnaire to 143 people from the Swiss Pediatric Renal Registry (SPRR), who were alive, over 18 years old, started KRT before the age of 18 years, and were German speakers. We measured psychological distress using the Brief Symptom Inventory 18 (BSI-18) and evaluated the Global Severity Index 18 (GSI-18), reflecting the overall level of distress, and the three subscales: depression, somatization, and anxiety. We compared levels of psychological distress to normal data from the Swiss general population and used regression models to identify associations with sociodemographic and clinical characteristics.

**Results:**

Eighty persons with a mean age of 39 years (SD 10.1) responded to the questionnaire (response rate 56%). Overall, the GSI-18 and all subscales of the BSI-18 were similar. Unemployed participants (25%) reported higher levels of somatization and were more likely to experience psychological distress. Participants using psychotropic drugs (14%) reported higher levels of overall psychological distress (10%), depression (13%) and somatization (9%).

**Conclusions:**

Adults after KRT during childhood showed good long-term psychological well-being. These results are encouraging and underline the favorable outcome of these patients. So besides the excellent somatic outcome, these patients can achieve a psychological healthy life after diagnosis of chronic kidney disease.

**Graphical abstract:**

**Figure:**
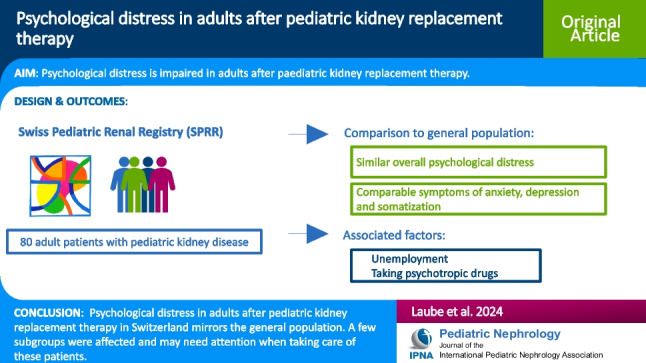
A higher resolution version of the Graphical abstract is available as Supplementary information

**Supplementary Information:**

The online version contains supplementary material available at 10.1007/s00467-024-06571-7.

## Introduction

Kidney replacement therapy (KRT) for children and adolescents with chronic kidney disease steadily developed during the last decades and patient and graft survival rates improved significantly [1, 2]. More recently, somatic long-term side effects following KRT had been recognized as a major burden [3]. Current treatment strategies aim to minimize well-known long-term complications such as cardiovascular diseases, malignancies, and growth retardation [3]. Despite those side effects, KRT seems to be an optimal treatment strategy with favorable somatic long-term outcome accompanied by good health-related quality of life (HRQoL) [4, 5]. As the survival rate has improved considerably in recent years and many children and adolescents are reaching adulthood, more attention towards their adult life is needed. In a Swiss cohort, we recently assessed HRQoL of adults, who had started KRT during childhood. Participants reported lower physical, but similar mental HRQoL in comparison to the general population. However, we found that they were more likely to experience adverse social and professional outcomes [5, 6].

There have been several studies on psychological distress, which can be defined as a set of painful mental and physical symptoms [7], in children and adolescents, both before, during and after transplantation [7–12]. Other publications have investigated the impact of other chronic diseases on psychological distress in children and adolescents (e.g., cancer, diabetes, liver transplantation, lung transplantation, heart transplantation) [13–15]. Additionally, psychological distress had also been studied in adults suffering from chronic kidney disease on dialysis or after being transplanted [16]. However, little is known about psychological distress in adults after successful pediatric kidney transplantation during childhood. We therefore aimed to describe psychological distress in adults from the Swiss Pediatric Renal Registry (SPRR) [17], who had received kidney replacement therapy during childhood. We aimed to compare the level of psychological distress with the Swiss general population and evaluated associations with sociodemographic and clinical characteristics.

## Methods

### Study population and design

We included individuals registered in the SPRR. The SPRR has existed since 1970 with the primary goal of collecting data on children with chronic kidney disease who underwent KRT [17]. For this study, we applied the following inclusion criteria: begin of KRT (kidney transplantation or initiation of chronic dialysis) before the age of 18 years; age over 18 years; and, alive at the start of study and initially treated in a pediatric hospital in the German speaking part of Switzerland [5]. We included only patients from the German speaking part of Switzerland due to limited financial resources [6].

We sent participants a postal questionnaire between 2021 and 2022. Therefore, we updated the postal addresses of the patients via the national postal service and contacted the hospitals, where patients were transferred after leaving pediatric care [5]. Participants were given the option to respond either online or in paper format. We sent two reminders: one after one month and another two months after the initial mailing. The questionnaire was based on a pilot study of the SPRR and a survey by the Swiss Childhood Cancer Survivor study [18]. It included questions about kidney disease, including its type, age at onset and number of transplants. In addition, we collected data on HRQoL, psychological well-being, health behaviour, education, occupation, current medication, and other physical illnesses [5].

### Evaluation of psychological distress

In our study, we used the short version of the Brief Symptom Inventory (BSI), the BSI-18 [19, 20]. The BSI-18 showed good validity and psychometric properties in several studies [21, 22]. The BSI-18 comprises 18 items selected from the original 53 items of the BSI [19]. All these 18 items collectively form the Global Severity Index 18 (GSI-18). The BSI-18 includes three symptom subscales: Somatization, Depression, and Anxiety (6 items for each scale). The BSI assesses the perceived impairment caused by each symptom over the last seven days, with response options ranging from “Not at all” (0) to “Very strongly” (4) [19]. If a participant did not respond to one item within a scale, the missing item was imputed with the mean value of the available items, as previously done [7]. If more than one item per scale was missing, this person's data was excluded [18]. Three different sum scales can be calculated from the BSI-18: the GSI-18 (Global Severity Index), reflecting the overall level of distress; the Positive Symptom Total (PST), indicating how many items were answered positively (i.e., at least 1 point on the scale of 0 to 4); and the Positive Symptom Distress Index (PSDI), which indicates the distress of these symptoms [19]. The PSDI is calculated by dividing the GSI sum score by the PST. The sum scores for the GSI-18, Somatization, and Depression and Anxiety were converted to T scores (Mean = 50, SD = 10) [19]. Severe psychological distress is defined as a T score ≥ 63, which is above the 90th percentile of the normal population [19].

### Sociodemographic and clinical variables

We collected the following sociodemographic and clinical variables in the questionnaire [5, 6]: Sociodemographic factors included gender (female/male), age (years), relationship status (being in a relationship: no/yes), living situation (living alone/living with others), occupational status (employed or studying/unemployed); and having children (no/yes). We obtained the following clinical variables from the SPRR: type of kidney disease categorized into congenital anomalies of the kidney and urinary tract (CAKUT), monogenetic diseases, and acquired diseases, age at start of KRT (< 10 years or ≥ 10 years) and duration of the disease (< 25 years or ≥ 25 years). We assessed the total number of transplants, current therapy for kidney disease (dialysis/transplant) and current medication, specifically focusing on psychotropic drugs, defined as antidepressants and sleep medication (no/yes).

### Statistical analysis

We used STATA 16.1 (StataCorp LP, College Station, TX) to analyse our data. We calculated the three sum scales of the BSI-18 and the sum value of the GSI-18. We assessed the reliability of individual subscales, by computing Cronbach's alpha. Then we determined standardized T scores for individual subscales and the GSI-18 (mean T score = 50, SD = 10). We tested standardized T scores for normal distribution using the Shapiro–Wilk test.

We first compared our mean sum scores with those of the Swiss general population using two-sided t-tests, both overall and stratified by gender [7]. We determined the proportion of participants suffering from severe psychological distress (T score ≥ 63) and compared it with the expected value of 10% from the normal population [19]. We used univariable linear regression models adjusted for age and gender to evaluate associations between T score of the GSI-18 and the three symptom scales and sociodemographic or clinical factors. We adjusted for age and gender, because they may affect levels of psychological distress [23, 24]. To explore the associations between severe psychological distress (T score GSI-18 ≥ 63) and clinical and sociodemographic factors we used univariable logistic regression models.

## Results

### Participants and their characteristics

**Fig. 1:**
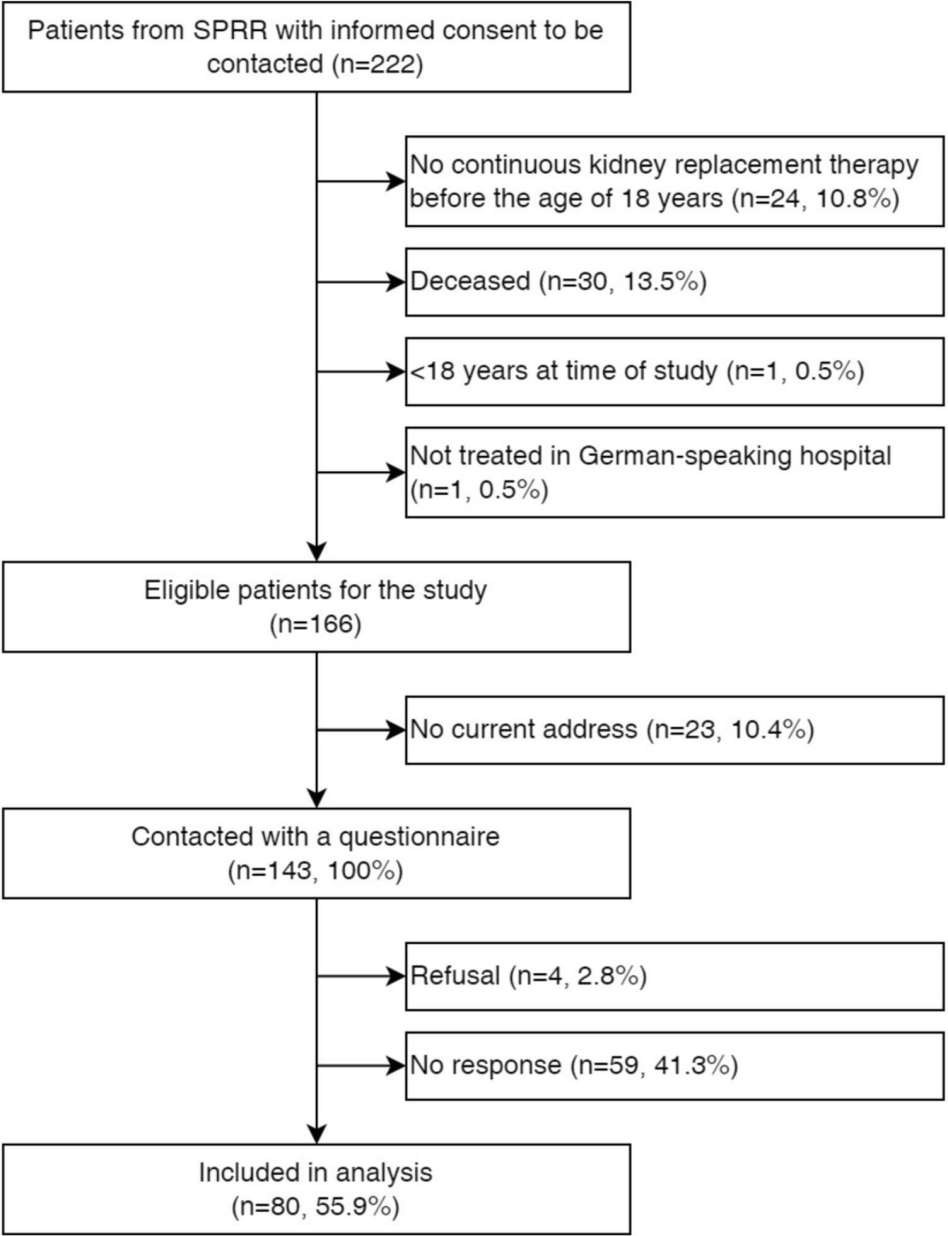
Flow chart of the study population [1]. SPRR, Swiss Pediatric Renal Registry

Of the 166 eligible participants, we contacted 143 with the questionnaire, and 80 answered the questionnaire (Fig. 1). The questionnaire was completed on paper by 66% and online by 34%. The mean age of the 80 participants was 39 years (SD 10.1) and 56% were male. During the questionnaire, all participants had a transplant, 84% had a functioning graft, 12% were on dialysis and in 4% no data was available (Table 1). Table 1 displays the clinical and sociodemographic characteristics of the study participants. A total of 25% of our study participants were unemployed, 14% were using psychotropic drugs, 86% did not have any children, 33% were living alone and 54% had no partner (Table 1). Five participants had one item missing in BSI-18 scales, but none had > 1 missing item. Our comparison group was derived from a survey including 1238 Swiss citizens, representing the Swiss general population [7]. Comparison data were collected between May 2015 and June 2016. The comparison sample was aged between 18 and 75 years (mean of 49 years, SD 15.2) and included 42% male and 58% female participants [7].

**Table 1:** Clinical and social characteristics of adults after kidney replacement therapy during childhood in Switzerland (N = 80)[2]

	n	%
*Mean age at study in years (range)*	39 (19–63)	
**Gender**		
Male	45	56
Female	35	44
**Type of kidney disease**		
Congenital anomalies kidney/ urinary tract	29	36
Monogenetic hereditary	35	44
Acquired	16	20
**Age at first KRT**		
Mean in years (range)	10.3 (0–17.9)	
< 10 years	32	40
≥ 10 years	48	60
**Duration of KRT**		
Mean in years (range)	28.3 (10.5–48.7)	
< 25 years	32	40
≥ 25 years	48	60
**Type of KRT at study**		
Transplantation	67	84
Dialysis	10	12
Missing	3	4
**Number of transplants**		
1	45	56
> 1	35	44
**Use of psychotropic drugs**^**a**^		
No	68	85
Yes	11	14
Missing	1	1
**Employment status**		
Employed or studying	60	75
Unemployed	20	25
**Partner/Relationship**		
Yes	36	45
No	43	54
Missing	1	1
**Living situation**		
Living alone	26	33
Not living alone	54	67
**Having children**		
Yes	11	14
No	69	86

KRT kidney replacement therapy

^*a*^*antidepressants and sleeping medication*

### BSI-18 scores and comparison to the Swiss general population

In our study population the mean sum score for the GSI-18 was 5.5 (95% Confidence Interval [95% CI]: 4.1–6.9) compared to 5.7 (95% CI: 5.3–6.1) in the general Swiss population (Table 2). Mean sum scores of the subscales somatization, depression, and anxiety were similar between study participants and the general Swiss population (all p > 0.05). Among our study participants (Supplementary Table 1), 10% had a T score ≥ 63 in the GSI-18 (female: 11%; male: 9%). A total of 10.2% participants in the Swiss general population were considered as cases with psychological distress (female: 10.5%; male 10.6%) [7]. In our study population the percentage of a T score ≥ 63 for depression was 13% (female: 14%; male: 11%; normative value: 9%). Nine percent had a T score ≥ 63 for somatization (female: 14%; male: 4%; normative value: 9%), while 11% had a T score ≥ 63 for anxiety (female: 6%; male: 16%; normative value: 9%).

**Table 2:** Psychological distress in adults after kidney replacement therapy (*N* = 80) and the Swiss general population (*N* = 1238): Comparison of mean sum scores of all BSI domains

	Adults after KRT			Swiss general population				
BSI Domain	*N*	Mean Score	95% CI	*N*	Mean Score	95% CI	T score difference	*p*-value
**Somatization**								
All	80	1.61	1.16–2.06	1238	1.63	1.50–1.75	- 0.09	0.93
Male	45	1.60	1.02–2.18	517	1.41	1.22–1.59	0.57	0.57
Female	35	1.63	0.89–2.37	721	1.83	1.65–2.01	- 0.52	0.61
**Depression**								
All	80	2.11	1.47–2.76	1238	1.94	1.76–2.12	0.50	0.62
Male	45	1.91	1.16–2.67	517	1.79	1.53–2.05	0.26	0.79
Female	35	2.37	1.21–3.53	721	2.08	1.84–2.32	0.49	0.63
**Anxiety**								
All	80	1.78	1.24–2.30	1238	2.15	1.99–2.31	- 1.32	0.19
Male	45	1.84	1.31–2.56	517	1.87	1.64–2.10	- 0.07	0.94
Female	35	1.69	0.84–2.53	721	2.40	2.18–2.62	- 1.60	0.12
**GSI-18**								
All	80	5.50	4.12–6.88	1238	5.71	5.32–6.10	- 0.29	0.77
Male	45	5.36	3.76–6.96	517	5.07	4.50–5.65	0.42	0.67
Female	35	5.69	3.19–8.18	721	6.31	5.68–6.84	- 0.49	0.63

*BSI* Brief Symptom Inventory, *CI *Confidence Interval, *GSI-18* Global Severity Index 18, *KRT* kidney replacement therapy

### Associations between sociodemographic and clinical variables and BSI-18

Using multivariable linear regression models, we found that unemployed participants had a 6.1 points (95% CI: 1.0–11.2) higher T score for somatization compared to those employed or in education. No other significant associations were observed between the BSI-18 scores and sociodemographic factors (Table 3).

**Table 3:** Associations between BSI-18 T scores sociodemographic and clinical variables in adults after kidney replacement therapy during childhood: Multivariable linear regression models (N = 80)

	GSI-18		Somatization		Depression		Anxiety	
	β (95% CI)	*p*-value	β (95% CI)	*p*-value	β (95% CI)	*p*-value	β (95% CI)	*p*-value
**Gender**								
Male (ref)								
Female	0.7 (-3.9, 5.3)	0.76	-0.1 (-4.6, 4.5)	0.98	1.9 (-2.7, 6.4)	0.42	-0.4 (-5.0, 4.2)	0.86
**Age (years)**	-0.1 (-0.3, 0.2)	0.60	0.1 (-0.2, 0.3)	0.56	-0.1 (-0.3, 0.1)	0.40	-0.1 (-0.3, 0.1)	0.40
**Employment status**								
Employed or studying (ref)								
Unemployed	2.9 (-2.3, 8.1)	0.28	**6.1 (1.0, 11.2)**	**0.02**	3.5 (-1.7, 8.6)	0.19	-1.8 (-7.1, 3.4)	0.49
**Relationship**								
Single (ref)								
In a relationship	-2.6 (-7.2, 2)	0.26	-0.1 (-4.7, 4.6)	0.98	-3.5 (-8.0, 1.1)	0.13	-2.5 (-7.1, 2.1)	0.27
**Living situation**								
Living alone (ref)								
Not living alone	-2.0 (-6.9, 2.9)	0.42	-2.5 (-7.4, 2.4)	0.31	-2.3 (-7.1, 2.6)	0.35	-0.35 (-5.2, 4.6)	0.89
**Children**								
No (ref)								
Yes	0.1 (-6.7, 6.9)	0.98	4.2 (-2.5, 10.9)	0.22	-0.7 (-7.5, 6)	0.83	-2.4 (-9.1, 4.4)	0.49
**Kidney disease**								
Congential kidney diseases^b^ (ref)								
Aquired kidney disease	4.7 (-1.1, 10.5)	0.11	-0.3 (-6.2, 5.6)	0.91	5.6 (-0.2, 11.3)	0.06	5.7 (-0.1, 11.4)	0.05
**Number of transplants**								
1 (ref)								
> 1	1.8 (-3.4, 7.1)	0.49	-0.4 (-5.6, 4.9)	0.89	1.75 (-3.5, 7)	0.51	2.9 (-2.3, 8.1)	0.27
**Duration of KRT (years)**	-0.2 (-0.7, 0.3)	0.37	-0.3 (-0.8, 0.2)	0.25	-0.2 (-0.7, 0.3)	0.40	-0.1 (-0.6, 0.4)	0.73
**Age at first KRT (years)**	0.2 (-0.3, 0.7)	0.37	0.3 (-0.2, 0.8)	0.25	0.2 (-0.3, 0.7)	0.40	0.1 (-0.4, 0.6)	0.73
**KRT at study**								
Transplant (ref)								
Dialysis	1.3 (-6.0, 8.5)	0.73	-1.2 (-8.3, 6)	0.74	2.7 (-4.4, 9.9)	0.45	1.0 (-6.2, 8.2)	0.78
**Use of psychotropic drugs**								
No (ref)								
Yes	**9.1 (2.4, 15.8)**	**0.01**	**9.5 (2.8, 16.2)**	**0.01**	**7.3 (0.6, 14.1)**	**0.03**	6.7 (-0.1, 13.6)	0.05

CI Confidence Interval, GSI-18 Global Severity Index 18, KRT kidney replacement therapy

^b^congenital anomalies of the kidney and urinary tract (CAKUT) and monogenetic diseases

We found that participants using psychotropic drugs had higher T scores for the GSI-18 (β = 9.1, 95% CI: 2.4–15.8), somatization (β = 9.5, 95% CI: 2.8–16.2), and depression (β = 7.3, 95% CI: 0.6–14.1). No associations with other clinical characteristics were identified (Table 3*)*.

Using the cut-off for severe psychological distress (T score GSI-18 ≥ 63), we found that unemployed persons were more likely to experience severe psychological distress compared to those who were employed or in education (odds ratio (OR) = 6.3, 95% CI 1.4–29.6). We observed that the association of psychotropic drugs was not significant when using the cutting of psychological logistic score, as the sample was smaller. No other associations with clinical and sociodemographic factors were identified (Supplementary Table 1).

## Discussion

This study found that adults after KRT during childhood in Switzerland did not have increased psychological distress compared to the general population. Only 10% of the study population had clinically relevant levels of psychological distress which is comparable to the Swiss general population. This suggests that in Switzerland, adults who had received a kidney transplant during childhood are not at increased risk for depression, somatization, or anxiety. Adults after KRT during childhood, who were unemployed and those treated with psychotropic drugs reported higher levels of psychological distress.

Comparing our data with other studies is challenging, as the patient population varies between studies in terms of medical situation (chronic kidney disease, dialysis or transplantation) and age at follow-up (children, adolescents or adults). Many previous studies focused on children and adolescents. A study including 28 patients aged 9–18 years with chronic kidney disease stage 1–4, not yet dialyzed or transplanted, reported more separation anxiety and depressive symptoms compared to healthy children, but resilience was similar in both groups [10]. An Egyptian study including 19 dialyzed children observed a high prevalence of psychiatric disorders (68.4%) and depression (26.3%) [8]. In a Peruvian evaluation of 67 children and adolescents (age 8–18 years) on dialysis, depressive symptoms occurred more frequently in females and were associated with weekly K_t_/V. But the type of dialysis showed no association with the occurrence of depressive symptoms [9]. This contrasts with our results, where there was no difference between male and female participants. However, comparability with our study is limited by different methods, outcome definitions, and the older age of our cohort.

Only a few studies have focused on transplanted children and adolescents. An American study of 44 children aged 9–18 years found significant symptoms of depression in 30% with higher scores among patients aged ≥ 13 years. However, the study population was too small to compare different groups of kidney replacement status, although longstanding kidney disease was declared to be a risk factor for depression [12]. A very high depression rate of 64% in children and adolescents with various stages of KRT was described by Cuellar et al. [25], whereas another study indicated a high prevalence of sleep disturbances ranging from 77 to 85% in children with KRT [11].

Taken together, there is some evidence in the literature that children with chronic kidney disease might express more symptoms of depression and therefore suffer from psychological distress. Our study indicates that early psychological problems might normalize with time after KRT.

As far as we know our study is the first to analyze psychological distress using the BSI in adults who had undergone kidney replacement therapy during childhood in Switzerland. Two studies found that adults suffer from various states of depression when being dialyzed or after kidney transplantation depending on type of dialysis, type of transplant and duration of disease [26, 27]. At a median age of 25.7 years, 25 out of 42 adults after pediatric transplantation stated that they felt anxiety or were depressed in a study by Kärrfelt et al*.* [28]. On the other hand, it was reported that psychological distress and post-traumatic stress disorder could result in “post traumatic growth”, defined as a positive and motivating psychological chance leading to positive coping strategies [16].

Comparisons with studies including other patient populations with childhood onset health conditions are difficult due to the inherent differences in the severity, prognosis, and long-term outcomes. A study including adolescents after heart, lung or liver transplantation indicated impairment in several areas such as anxiety, disappointment, or social adjustment. The individuals also reported the obligation to show gratitude towards the donor, pressure to take good care of the transplant and, in the case of a living donation, fear for the donor due to the possible health risks [13]. Also, children with type 1 and 2 diabetes seem to experience increased anxiety and depression [15]. Swiss childhood cancer survivors, also evaluated using the BSI, indicated a lower T score in the GSI, in obsessive–compulsive tendencies, anxiety and somatization compared with the German-speaking normal population. However, it was found that participants had a higher risk of suffering from severe psychological stress in comparison to the normal population [29]. Comparing these results with our study population indicates the difference between cancer survivors and patients with KRT. Cancer survivors usually are cured of their disease and might try to leave it behind, while individuals after kidney transplantation face a lifelong dependency on regular medical check-ups, medication to prevent graft rejection and a high probability of needing additional KRT again later in life.

A limitation of our study is the relatively small sample size limiting statistical power for subgroup comparisons, especially regarding clinical and sociodemographic characteristics. Additionally, our study included only German-speaking patients, so results may not be representative for all of Switzerland. In the comparison group all language regions of Switzerland were included, which may limit comparability [7]. Furthermore, data were collected between 2021 and 2022 whereas data from the general Swiss population were obtained between 2015 and 2016. Differences caused by broader circumstances (e.g., the COVID-19 pandemic) could not be avoided or accounted for.

The strength of our study is the use of a representative comparison sample from the Swiss general population that completed the same questions on psychological distress. Further, our study included adult patients after KRT during childhood with an average age of 39 years, providing long-term follow-up information on this population. Another strength refers to the use of high-quality clinical information based on medical records from the SPRR. If we compare our response rate with that of other studies, we must take into account the circumstances in which people were contacted. In most studies, children were interviewed during their KRT [8–10, 12]. In the paper by Kärrfelt et al., 42 of the 68 contacted young adults after KRT during childhood took part in the survey (response rate 61%). Our response rate of 56% seems to be sufficient for statistical analysis [28]. In comparison to other studies, our study sample is large and covers an extensive long-term follow-up period, thereby enriching the current literature.

Chronic kidney disease in children and adults does have a massive long-term impact on patients which leads to a life-long dependence on medical support. This may intuitively interfere with their psychological wellbeing and affect social, educational, and family life. Our results therefore might surprise, but underline the fact that kidney transplantation programs in children and adolescents are successful not only in terms of survival of patient and graft, controllable side effects of the treatment and HrQoL, but also in terms of psychological distress. Although carrying the burden of a chronic disease, adults after KRT during childhood appear to be psychologically well-adjusted in Switzerland. The Swiss care system for these patients does follow a multidisciplinary and multiprofessional concept including pediatric nephrologists, specialized nurses, psychologists, social workers, dieticians and school teachers. Patients were also following a structured transition program, as described earlier [30].

Our results should encourage pediatric nephrologists and the whole caring team to sustain their daily efforts treating these patients. Furthermore, it should motivate adult nephrologists who care for them later in adulthood.

In conclusion, our study indicates similar levels of psychological distress in 80 adults after pediatric kidney replacement therapy in comparison to the Swiss general population. Our data are encouraging for pediatric nephrologists and generally indicate favorable psychological outcomes in the long-term. Beside the well-known excellent somatic outcome, these patients can achieve a psychologically healthy life many decades after diagnosis of chronic kidney disease.

## Supplementary Information

Below is the link to the electronic supplementary material.

Graphical abstract (PPTX 596 KB)

Supplementary file2 (DOCX 24 KB)

## Data Availability

The data that support the information of this manuscript were accessed on secured servers of the Institute of Social and Preventive Medicine at the University of Bern. Data can only be made available for researchers who fulfil the respective legal requirements. All data requests should be communicated to the corresponding author.

## Abbreviations

BSI: Brief Symptom Inventory
CAKUT: Congenital Anomalies of the Kidney and Urinary Tract
CI: Confidence Interval
GSI: Global Severity Index
HRQoL: Health-Related Quality of Life
KRT: Kidney Replacement Therapy
PSDI: Positive Symptom Distress Index
PST: Positive Symptom Total
SCL-90: Symptom Checklist-90
SPRR: Swiss Pediatric Renal Registry
