# Supplementary material for: Psychological distress in adults after pediatric kidney replacement therapy
Source: Pediatr Nephrol. 2024 Nov 5;40(4):1049–57. doi: 10.1007/s00467-024-06571-7 (PMC11885388; doi:10.1007/s00467-024-06571-7)
Supplement: Supplementary file 1 — Graphical abstract (PPTX 596 KB) [file 467_2024_6571_MOESM1_ESM.pptx]

## Slide 1
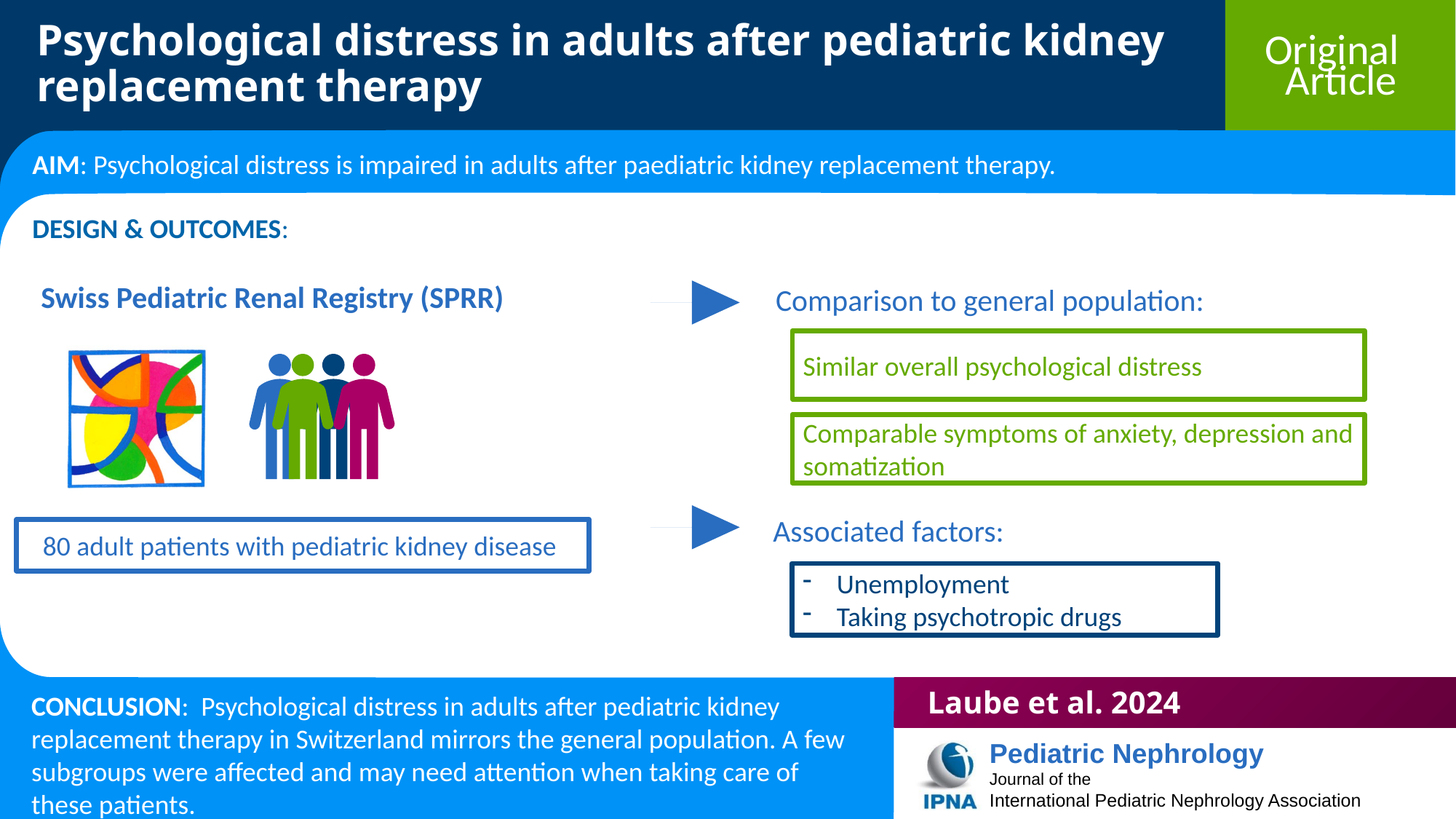

Psychological distress in adults after pediatric kidney replacement therapy
AIM: Psychological distress is impaired in adults after paediatric kidney replacement therapy.
DESIGN & OUTCOMES:
Swiss Pediatric Renal Registry (SPRR)
Comparison to general population:
Similar overall psychological distress
Comparable symptoms of anxiety, depression and somatization
Associated factors:
80 adult patients with pediatric kidney disease
Unemployment
Taking psychotropic drugs
Laube et al. 2024
CONCLUSION: Psychological distress in adults after pediatric kidney replacement therapy in Switzerland mirrors the general population. A few subgroups were affected and may need attention when taking care of these patients.
