# Supplementary material for: Psychological distress in adults after pediatric kidney replacement therapy
Source: Pediatr Nephrol. 2024 Nov 5;40(4):1049–57. doi: 10.1007/s00467-024-06571-7 (PMC11885388; doi:10.1007/s00467-024-06571-7)
Supplement: Supplementary file 2 — Supplementary file2 (DOCX 24 KB) [file 467_2024_6571_MOESM2_ESM.docx]

## **Supplementary Table**

**Table 1: Associations between clinically relevant levels of psychological distress (T score GSI-18 > 63) and sociodemographic and clinical variables from univariable logistic regression models**

|  |  | Cases T score ≥ 63 | Univariate logistic Regression | | |
| --- | --- | --- | --- | --- | --- |
| Factor | Number | N (%) | OR | 95% CI | *p*-value |
| **Sex** |  |  |  |  |  |
| Male | 45 | 4 (9) | ref |  |  |
| Female | 35 | 4 (11) | 1.3 | 0.3-5.7 | 0.71 |
| **Age** |  |  |  |  |  |
| < 45 years | 59 | 5 (8) | ref |  |  |
| ≥ 45 years | 21 | 3 (14) | 2.3 | 0.2-24.9 | 0.50 |
| **Type of Kidney disease** |  |  |  |  |  |
| Congenital anomalies kidney/urinary tract | 29 | 1 (3) | ref |  |  |
| Monogenic hereditary diseases | 35 | 4 (11) | 3.6 | 0.4-34.3 | 0.26 |
| Acquired | 16 | 3 (19) | 6.5 | 0.6-68.2 | 0.12 |
| **Age at first KRT** |  |  |  |  |  |
| <10 years | 32 | 2 (6) | ref |  |  |
| ≥10 years | 48 | 6 (13) | 2.1 | 0.4-11.4 | 0.37 |
| **Duration of KRT at study** |  |  |  |  |  |
| ≥25 years | 48 | 4 (8) | ref |  |  |
| <25 years | 32 | 4 (13) | 1.6 | 0.4-6.8 | 0.55 |
| **Type of KRT at study** |  |  |  |  |  |
| Transplantation | 67 | 6 (9) | ref |  |  |
| Dialysis | 10 | 2 (20) | 2.5 | 0.4-14.8 | 0.29 |
| **Number of transplants** |  |  |  |  |  |
| 1 | 45 | 4 (9) | ref |  |  |
| >1 | 35 | 4 (11) | 1.3 | 0.3-5.7 | 0.71 |
| **Use of psychotropic drugs** |  |  |  |  |  |
| No | 68 | 6 (85) | ref |  |  |
| Yes | 11 | 2 (15) | 4.7 | 1.0-23.6 | 0.06 |
| **Employment status** |  |  |  |  |  |
| Employed or studying | 60 | 3 (5) | ref |  |  |
| Unemployed | 20 | 5 (25) | **6.3** | **1.4-29.6** | **0.02** |
| **Partnership/Relationship** |  |  |  |  |  |
| No | 43 | 6 (14) | ref |  |  |
| Yes | 36 | 2 (6) | 2.8 | 0.5-14.6 | 0.23 |
| **Living situation** |  |  |  |  |  |
| Living alone | 54 | 5 (9) | ref |  |  |
| Not living alone | 26 | 3 (12) | 1.3 | 0.3-5.8 | 0.75 |
| **Having children** |  |  |  |  |  |
| Yes | 11 | 1 (9) | ref |  |  |
| No | 69 | 7 (10) | 1.1 | 0.1-0.8 | 0.91 |

CI, Confidence Interval; KRT, kidney replacement therapy
